# Diosgenin Glucoside Protects against Spinal Cord Injury by Regulating Autophagy and Alleviating Apoptosis

**DOI:** 10.3390/ijms19082274

**Published:** 2018-08-02

**Authors:** Xian-Bing Chen, Zi-Li Wang, Qing-Yu Yang, Fang-Yu Zhao, Xiao-Li Qin, Xian-E Tang, Jun-Long Du, Zong-Hai Chen, Kui Zhang, Fei-Jun Huang

**Affiliations:** 1 West China School of Basic Medical Sciences and Forensic Medicine, Sichuan University, Chengdu 610041, China; 1998023@hbmy.edu.cn (X.-B.C.); zhk@scu.edu.cn (K.Z.); 2 College of Medicine, Hubei University for Nationalities, Enshi 445000, China; wangzili2018731@gmail.com (Z.-L.W.); yangqingyuyqy@gmail.com (Q.-Y.Y.); fangyuzhao584@gmail.com (F.-Y.Z.); xiaolitan965@gmail.com (X.-L.Q.); tangx483@gmail.com (X.-E.T.); dujunlong666@gmail.com (J.-L.D.); chenzonghai666@gmail.com (Z.-H.C.)

**Keywords:** spinal cord injury, autophagy, diosgenin glucoside, Rheb/mTOR signal pathway, miR-155-3p, apoptosis

## Abstract

Spinal cord injury (SCI) is a severe traumatic lesion of central nervous system (CNS) with only a limited number of restorative therapeutic options. Diosgenin glucoside (DG), a major bioactive ingredient of *Trillium tschonoskii* Max., possesses neuroprotective effects through its antioxidant and anti-apoptotic functions. In this study, we investigated the therapeutic benefit and underlying mechanisms of DG treatment in SCI. We found that in Sprague-Dawley rats with traumatic SCI, the expressions of autophagy marker Light Chain 3 (LC3) and Beclin1 were decreased with concomitant accumulation of autophagy substrate protein p62 and ubiquitinated proteins, indicating an impaired autophagic activity. DG treatment, however, significantly attenuated p62 expression and upregulated the Rheb/mTOR signaling pathway (evidenced as Ras homolog enriched in brain) due to the downregulation of miR-155-3p. We also observed significantly less tissue injury and edema in the DG-treated group, leading to appreciable functional recovery compared to that of the control group. Overall, the observed neuroprotection afforded by DG treatment warrants further investigation on its therapeutic potential in SCI.

## 1. Introduction

Spinal cord injury (SCI) remains a rare disease in comparison to traumatic brain injury, concussion, or stroke in the younger population, but it can cause permanent disability or loss of movement and sensation. It can lead to a series of complications with complex pathological process, and more difficulties in rehabilitation, with only a limited number of therapeutic options to improve functional recovery. In China, the incidence of SCI is increasing yearly [1]. Therefore, SCI repair is a hot topic in clinical research.

At present, the pathological and repairing mechanisms of SCI mainly focuses on the prevention and reversible regulation of secondary injury of spinal cord, which will provide reliable clinical treatment strategies for the recovery and regeneration of neurons after SCI. The initial trauma is followed by complex, poorly understood changes resulting in further destruction of the spinal cord tissue over a period of several months, and consequently loss of the neural function. The apoptosis of neurons is an important mechanism in the pathogenesis of SCI [2,3]; therefore, the major strategy is to reduce neuronal damage, inhibit apoptosis, and to control inflammatory responses for promoting spinal cord repair. In recent years, the regulatory role of autophagy in SCI has become a popular area of research. Zhang et al. [4,5,6,7] have documented that autophagy is beneficial to the recovery of neuron function after SCI through eliminating, degrading, and digesting damaged, denatured, aged, or dysfunctional cells via autophagy, thereby completing the reconstruction, regeneration, and repair of the cells to provide raw materials for achieving cell recycling. Chandler et al. [8,9,10] have reported that excessive autophagy promotes cell death during SCI progression, which is detrimental to neuron cells. Therefore, autophagy not only plays an important role in cell death, but also maintains cell homeostasis to protect cells in the absence of nutrients after SCI. In addition, endogenous non-coding microRNAs (miR) have also been found to have the function to regulate autophagy through modulating autophagy-related proteins. MicroRNAs, a class of non-coding tiny RNAs with lengths of 21–25 nucleotides, are widely involved in proliferation, differentiation, metabolism, development, apoptosis, and other complex physiological and pathological processes of eukaryotes [11,12]. In addition, endogenous microRNAs also have the function to regulate autophagy through modulating autophagy-related proteins. During SCI progression, many microRNAs have been specifically differentially expressed to execute the important regulatory functions for injury repairing [13,14]. Therefore, it is the focus of current biological research to clarify the regulatory mechanisms of microRNAs involved in the repairing of SCI. As a multifunctional microRNA, microRNA-155 (miR-155) presents overexpression in lung, heart and kidney [15]. Moreover, miR-155 can bind to the 3′-untranslated region (UTR) of the target genes such as Ras homolog enriched in brain (Rheb) and serine/threonine kinase p70S6 kinase (p70S6K), thereby negatively regulating autophagy in the body. Rheb as a small guanine triphosphate (GTP) enzyme belonging to the guanosine-binding protein Ras superfamily is widely present in a variety of eukaryotes. Increased expression of miR-155 will negatively inhibit the expression of Rheb, thereby accelerating the maturation of phagocytic bodies and autophagy [16,17,18]. As a highly conserved protein, Rheb plays an important role in cell growth, proliferation, cell cycle, apoptosis, and autophagy [19]. Substantial data suggests that an increased expression level of Rheb can over-activate mTORC1 because Rheb is the upstream of mTORC1 and plays an important role in the regulation of mTORC1 activity [20,21]. Therefore, with the exploration of the correlation between microRNAs the and spinal cord, and corresponding regulatory mechanisms of microRNAs, microRNAs have a high potential as targets or drug mimics of SCI therapy, which will provide novel therapeutic strategies for SCI.

Thus far, a variety of treatment strategies have contributed little to the improvement of neurologic deficits in the SCI. We have previously determined that cell apoptosis is involved in the process of SCI, and have further studied the effects of diosgenin glucoside (DG) in the rat model of SCI. However, the clinical applications of these therapies are still limited. Therefore, exploring bioactive ingredients with low toxicity and side effects from natural products is an inevitable trend of drug development. DG is a major bioactive ingredient of *Trillium tschonoskii* Max. [22], with anti-cancer, anti-inflammatory, analgesic, immune-enhancing, cardiovascular function-promoting, blood pressure-reducing, and anti-aging functions [23,24,25]. Moreover, DG has also been shown to have a protective role in SCI through the up-regulation of ciliary neurotrophic factor (CNTF) and CNTF receptor α (CNTF-Rα) expression in spinal cord tissue [26], and can rescue dysfunctional autophagy to execute anti-aging through up-regulating Rheb and down-regulating mTOR [27] in our previous studies. However, it’s unclear whether DG treatment could protect against SCI through Rheb/mTOR signal pathways in vitro and in vivo. In this study, we aimed to evaluate the effect of DG on post-injury recovery of motor function and neuronal apoptosis in rats with experimental SCI, and we determine whether autophagy induced by miR-155-3p/Rheb/mTOR signal pathway is involved in the protection of SCI and in the repair of damaged neurons.

## 2. Results

### 2.1. DG Decreased the Structural Damage of Spinal Cord Tissue and Promoted Functional Recovery after SCI

**Figure 1:**
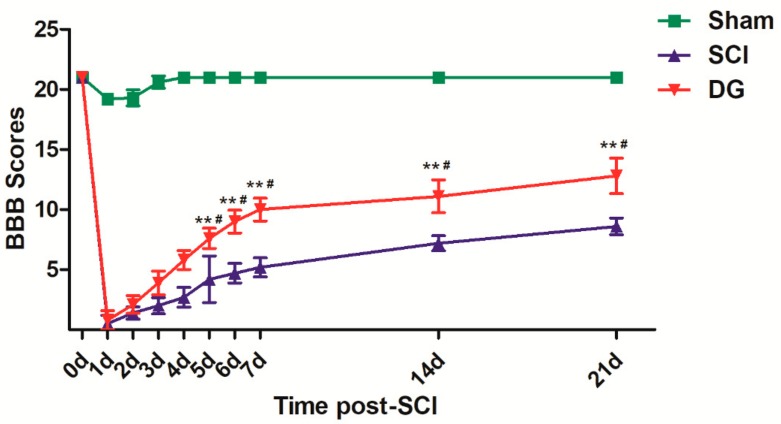
Neurological function measured by the Basso, Beattie, and Bresnahan (BBB) locomotion scores from 0 to 21 days after Spinal cord injury (SCI). In general, functional recovery gradually improved both in diosgenin glucoside (DG) and SCI groups from the fourth day after SCI. Except for the first four days the experimental period, the DG group showed significantly improved BBB scores in comparison with the SCI group. ** *p* < 0.01 in the DG group when compared with the sham group. # *p* < 0.05 in the DG group when compared with the SCI group (*n* = 10 per group).

To evaluate the therapeutic effect of DG on locomotor recovery after SCI, functional recovery was evaluated after injury for 21 days using Basso, Beattie, and Bresnahan (BBB) scores. BBB scores of the sham group were about 21 normally, whereas the BBB scores of SCI group and DG-treated group were below the normal score after injury (*p* < 0.01) (Figure 1). There was no significant difference in BBB scores between SCI group and DG-treated group after SCI for four days; however, BBB scores revealed an increase after the contusion with DG treatment from the fifth day when compared with SCI model group (*p* < 0.05). These data indicate that DG may influence the functional improvement of locomotor activity after SCI.

**Figure 2:**
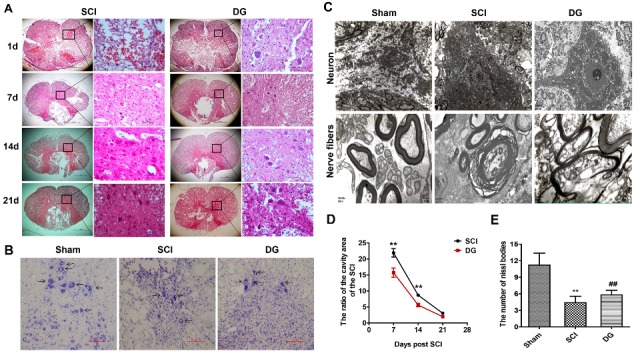
DG decreased the damage of tissue structure and the loss of neurons after experimental acute traumatic SCI. (**A**) HE staining at one, seven, 14, and 21 days. Scale bars are 10 μm (40×) and 100 μm (400×). T9-11, right anterior spinal cord; (**B**) Nissl staining for evaluating the loss of neurons at seven days. T9-11, spinal anterior horn motor neuron; Nissl staining positive cell ↑; scale bars are 20 μm (200×); (**C**) transmission electron microscopic image for evaluating the damage of tissue structure at seven days, T9-11, spinal anterior horn motor neurons and nerve fibers. The scale bars are 0.5 μm; (**D**,**E**) graphic presentation indicates the ratio of cavity area (cavity area, spinal cord cross-sectional area), the number of surviving neurons and the number of Nissl bodies. All data were expressed as M ± SD. ** *p* < 0.01 in the DG group when compared with the sham group. ## *p* < 0.01 in DG group when compared with the SCI group (*n* = 5 per group).

The difference in morphology of tissues from sham, SCI, and DG groups was analyzed by Hematoxylin-Eosin (HE) staining after the injury for one, seven, 14, and 21 days (Figure 2A). The ratio of the cavity area in the spinal cord cross-sectional area by HE staining was calculated somewhat, to histologically show the function of the spinal cord [28,29]. Severe damage of the dorsal white matter and central grey matter was observed in SCI rats when compared with the sham group. In contrast, obvious therapeutic effects were observed in DG-treated group, with a decreased cavity in the dorsal white matter and central gray matter, as compared with SCI group (*p* < 0.01) (Figure 2D).

Neurons stained with Nissl were counted to examine the effect of DG on the loss of motor neurons in the ventral horn. The number of Nissl bodies was increased in the DG group when compared with that of the SCI group (*p* < 0.01) (Figure 2B,E). In order to determine the morphological changes of the spinal cord and the structures of spinal cord from a sham operation, the SCI model, and DG treatment groups were examined by transmission electron microscopy (TEM). In the normal group, myelin sheaths were arranged in the integral structures and clear mitochondria. In comparison, the spinal cord in the SCI group revealed irregular-shaped, disordered structures with loose, twisted, wrinkled, or partially ruptured plates. Swollen and ruptured mitochondria were also observed in this group, with a greatly decreased number of mitochondria. However, in the DG group, myelin sheaths exhibited more regular shapes and integral structures, more uniformed lamellar structures, reduced degrees of loosening, improved degrees of rupture, and alleviated mitochondrial swelling, when compared with the SCI group (Figure 2C).

### 2.2. DG Attenuated Apoptosis Caused by SCI

**Figure 3:**
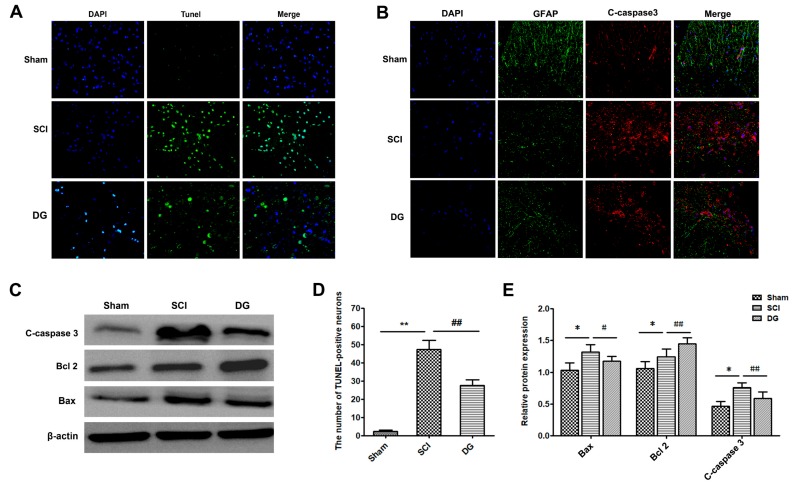
DG-attenuated apoptosis caused by SCI. (**A**,**D**) Immunofluorescence images of terminal-deoxynucleotidyl transferase (TdT)-mediated nick end labeling (TUNEL) (green) staining. The nuclei (blue) were labeled with DAPI (4′,6-diamidino-2-phenylindole). The data revealed the quantification of apoptotic cells. Scale bars are 10 μm; (**B**) immunofluorescence staining of caspase-3 protein (red), GFAP (glial fibrillary acidic protein) (green) and DAPI-labeled nucleus (blue). Scale bars are 10 μm; (**C**,**E**) representative Western blots and quantification data of Bax, BCL-2, caspase-3, and β-actin in each group. Columns represent M ± SD for all data. * *p* < 0.05 and ** *p* < 0.01 in the DG group when compared with the sham group. # *p* < 0.05 and ## *p* < 0.01 in DG group when compared with the SCI group (*n* = 5 per group).

In order to evaluate DG-modulated cellular apoptosis, the expression levels of cleaved caspase-3, Bax, and Bcl-2 were analyzed by Western blot and immunohistochemistry (IHC). Western blot analysis of cleaved caspase-3 showed that DG significantly reversed the increased level of cleaved caspase-3 caused by SCI (Figure 3B,E). Similarly, the expression of pro-apoptotic protein Bax was down-regulated, while the anti-apoptotic protein Bcl-2 was up-regulated after treatment with DG (Figure 3C,E). Moreover, terminal-deoxynucleotidyl transferase (TdT)-mediated nick end labeling (TUNEL) staining of the spinal cord was performed to estimate the amount of apoptotic cells within in each group. As shown in Figure 3A,D, the number of apoptotic cells was increased significantly in the SCI group when compared with that of the sham group. In contrast, the number of TUNEL-positive neurons in the DG group was less than that of the SCI group (*p* < 0.01). Taken together, these results demonstrate that DG administration is an effective therapeutic strategy for the inhibition of apoptosis after SCI.

### 2.3. DG Down-Regulated miR-155-3p in Spinal Cord Tissue after SCI

**Figure 4:**
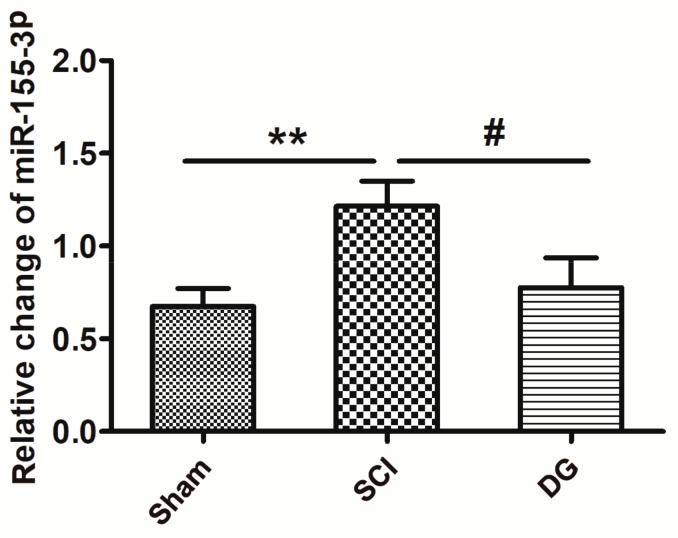
The miR-155-3p was activated in spinal cord tissue from SCI rats. The expression of miR-155-3p in different groups was analyzed through quantitative real-time polymerase chain reaction (qRT-PCR). All data were expressed as M ± SD. ** *p* < 0.01 in the DG group when compared with the sham group. # *p* < 0.05 in the DG group when compared with the SCI group (*n* = 5 per group).

By using a bioinformatics analysis, we examined two subtypes of miR-155, and the subtype miR-155-3p had altered expression after SCI. The expression of miR-155-3p was further confirmed by quantitative real-time polymerase chain reaction (qRT-PCR) analysis using the comparative Ct (ΔΔCt) method. The expression of miR-155-3p in spinal cord tissue was significantly different between groups. As shown in Figure 4, the expression of miR-155-3p was significantly increased in the SCI model group when compared with the sham group (*p* < 0.05). However, DG administration markedly mitigated the up-regulation of miR-155-3p after SCI (*p* < 0.05), suggesting that the up-regulation of miR-155-3p is associated with the SCI of rats.

### 2.4. DG-Activated Rheb/mTOR Signal Pathway after SCI

**Figure 5:**
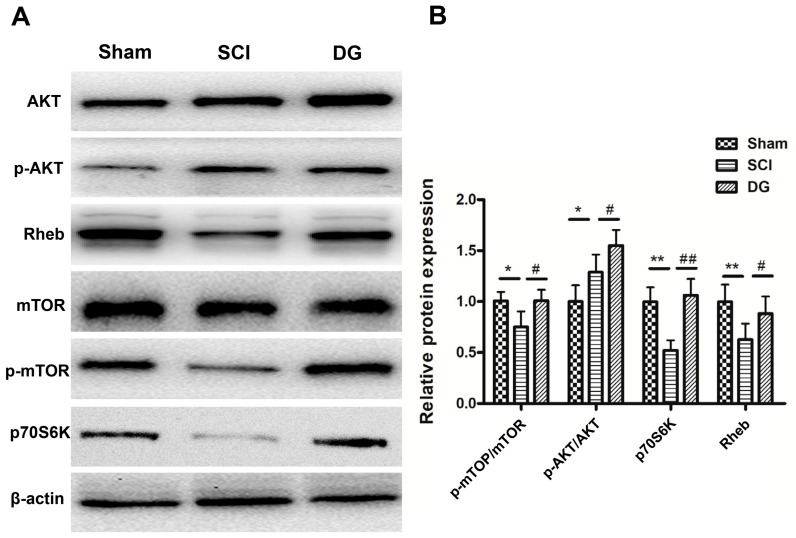
DG-activated Rheb/mTOR signal pathway after SCI. (**A**,**B**) Representative Western blots and quantification data of pAKT, AKT, p-mTOR, mTOR, p70S6K, Rheb, and β-actin in each group. All data were expressed as M ± SD. * *p* < 0.05 and ** *p* < 0.01 in the DG group when compared with the sham group. # *p* < 0.05 and ## *p* < 0.01 in the DG group when compared with the SCI group (*n* = 5 per group).

To explore whether the Rheb/mTOR signal pathway was activated by DG, the expression of Rheb, mTOR, p-mTOR, AKT, p-AKT, and p70S6K was detected in spinal cord tissues from each group by Western blot analysis after SCI for seven days (Figure 5A,B).

Rheb was significantly decreased, and mTOR and p70S6K was significantly reduced in spinal cord tissues from the SCI model group when compared with those in the sham group. DG treatment significantly up-regulated Rheb and p70S6K, and increased p-mTOR/mTOR ratio when compared with those in the SCI group. This result suggests that DG further activated the Rheb/mTOR signal pathway after SCI.

### 2.5. DG Rescued Abnormal Autophagy in Spinal Cord Tissues of SCI Rats

**Figure 6:**
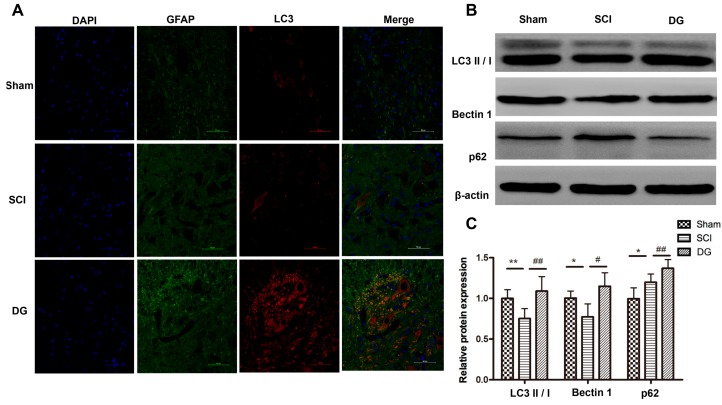
DG rescued the impaired autophagy in spinal cord tissues of rats with SCI. (**A**) Immunofluorescence staining of LC3 (red), GFAP (green) and DAPI-labeled nuclei (blue). Scale bars are 10 μm; (**B**,**C**) representative western blots and quantification data of LC3, Beclin1, p62 and β-actin in each group. All data were expressed as M ± SD. * *p* < 0.05 and ** *p* < 0.01 in the DG group when compared with the sham group. # *p* < 0.05 and ## *p* < 0.01 in the DG group when compared with the SCI group (*n* = 5 per group).

**Figure 7:**
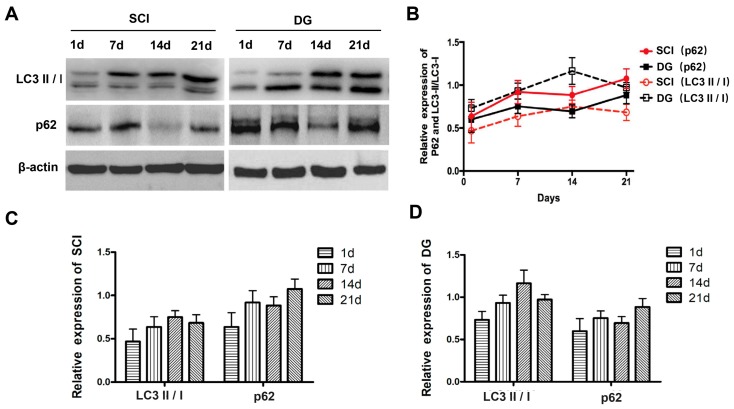
The anti-apoptosis effect of DG is related to cellular autophagy flux after SCI. (**A**–**D**) Representative Western blots and quantification data of p62, LC3II/LCI, and β-actin in each group. All data were expressed as M ± SD.

In order to verify the regulatory role of DG in autophagy, the expression of autophagy-associated proteins such as the ratio of LC3-II to LC3-I, Beclin1, and p62 was evaluated by Western blot and IHC. SQSTM1/p62, a polyubiquitin binding protein that is selectively incorporated into autophagosomes through direct binding to LC3 and efficient degradation during autophagy, was also examined. Thus, the total cellular levels of SQSTM1 reflect the extent of autophagy. The results showed that the ratio of LC3-II to LC3-I and the expression of Beclin1 were significantly decreased, and the expression of p62 was significantly increased in spinal cord tissue of SCI rats when compared with those in the sham group. However, the ratio of LC3-II to LC3-I and the expression of Beclin1 increased, while the expression of p62 decreased in DG group when compared with the SCI group. This suggests that dysfunctional autophagy in spinal cord tissue of SCI rats can be rescued by DG treatment (Figure 6A–C). To further analyze the degree of autophagy in the SCI and DG groups at each designated time point (one, seven, 14, or 21 days), the expression of p62 and LC3 was evaluated by western blot. The expression levels of p62 and LC3 were significantly increased in a time-dependent manner at one, seven and 14 days, respectively, but decreased at 21 days in DG group when compared with the SCI group (Figure 7A–D). These data suggest that autophagy is a dynamic process. DG treatment resulted in the reduction of p62 as compared with the SCI group, suggesting that DG increases autophagy by promoting degradation by autophagy.

## 3. Discussion

SCI is a more important type of injury in clinical practice, and it is an important topic of clinical research in the field of medicine. At present, the pathological and repairing mechanisms of SCI mainly focus on the prevention and reversible regulation of secondary injury of spinal cord, which will prevent further death of neurons and glial cells in spinal cord tissue, and will provide reliable clinical treatment strategies for the repairing and regeneration of neurons after spinal cord injury. The treatment using bioactive ingredients from traditional Chinese herbs with low toxicity and side effects has become a novel research trend. Previous studies have confirmed that DG has neuroprotective effects against SCI. According to reports in the literature, the apoptosis of neurons, oligodendrocytes, astrocytes, and microglia cells in tissues from injured segments and adjacent segments from 4 hr to 3 weeks could be observed after SCI [30,31,32]. The apoptosis of spinal cord neurons caused the impairment and loss of spinal cord sensory and motor functions [29]. Consistent with our findings, it is known that trauma and ischemia of the spinal cord can cause neuronal apoptosis, which could be attenuated by DG treatment in the present study. Our data also indicates that DG may influence the functional improvement of locomotor activity after SCI, as confirmed by Basso, Beattie, and Bresnahan (BBB) scores and TUNEL staining, which could be an effective therapeutic strategy for inhibiting apoptosis after SCI. In the spinal cord of patients with SCI, apoptosis was mainly found in the lesion and its surrounding tissues, while the Waller change was observed in the lesion center. Similarly, DG treatment can reduce loosening degree, improve rupture degree, and alleviate mitochondrial swelling, as observed by TEM when compared with the SCI group.

Previous studies have shown that microRNAs can play a broad range of roles in cell differentiation, proliferation, and apoptosis by regulating the expression of hundreds of genes via RNA-induced silencing complexes (RISC) and targeting RNA expression processes [33]. MicroRNAs are rich in the central nervous system, and reveal a close relationship with the development of neurological diseases, as well as widely influence the signaling networks, leading to pathological responses after SCI. MicroRNAs have gained extensive attention, and could become a novel therapeutic target for the treatment of SCI. A large number of studies have shown that miR-155 is a broad regulator of inflammatory mediators, and plays a key regulatory role in immune and inflammatory disorders [34,35,36,37]. The development of neurodegenerative diseases is closely correlated with immunity and inflammation, suggesting that miR-155 may be a novel target for its treatment. A report [15] has documented that there is high expression with more than 2-fold change of miR-155 and other microRNAs (miRNAs) in brain tissue with multiple lesion locations. We previously demonstrated that DG treatment could downregulate the expression of miR-155 in a rat model of D-galactose-induced brain senescence [30]. Based on these studies and bioinformatics analysis, we analyzed the expression of miR-155 by qRT-PCR, demonstrating that miR-155-3p was aberrantly expressed after SCI. In addition, our findings have implicated that DG treatment could down-regulate miR-155-3p expression after SCI, suggesting that miR-155-3p may play an important role in the pathogenesis of SCI. Therefore, it is necessary to verify the target gene of miR-155-3p during DG treatment after SCI in order to further explore the underlying mechanisms of DG.

Rheb is regulated by the PI3K signaling pathway, including PKB and TSC1/2. Rheb modulates the mTOR complex and controls protein synthesis via the phosphorylation of 4E-BP1 and S6. Wan et al. [38] had demonstrated that Rheb, RICTOR, and RPS6KB2 (p70S6K) of the mTOR signal pathway are direct targets of miR-155. miR-155 can bind to the 3′-UTR of the target genes *Rheb* and *p70S6K*, thereby negatively regulating autophagy. Our results demonstrated that DG treatment activated the Rheb/p70S6K signal pathway. In addition, Rheb is beneficial for the attenuation of neurodegenerative diseases including Alzheimer’s disease, Parkinson’s disease, and Huntington disease by secreting brain-derived growth factors (BDGF) and glial-derived neurotrophic factors, suggesting that Rheb plays an important role in the central nervous system. The overexpression of Rheb in the nervous system can promote the growth of axons [39] and the production of myelin after birth, which plays a protective role in neurons [40,41,42]. Rheb is the upstream member of mTORC1, and plays an important role in the regulation of mTORC1 activity [43]. Rheb can bind to GTP to promote cell growth and cell cycle regulation, and insulin signaling pathways play an important role in maintaining the growth of organisms. mTOR kinase plays an important role in the transduction of extracellular to intracellular signals, and is an important target for the treatment of neurological diseases. The function of mTOR can be accomplished through a complex of mTORC1 and mTORC2. Rheb regulates the self-renewal of neural stem cells by regulating the activity of mTORC1, which plays an important role in the development of the cerebral cortex. In contrast, in injured neurons, Rheb is mainly expressed in astrocytes and neurons [44,45], indicating that Rheb is highly correlated with the proliferation and apoptosis of neural progenitor cells. Our data showed that DG treatment could significantly increase p-mTOR/mTOR ratio, implying that DG could further activate Rheb/mTOR signal pathway and inhibit miR-155-3p expression after SCI. In order to confirm whether miR-155-3p is related to the expression of Rheb, we will detect the expression of Rheb/mTOR signal pathway using a miR-155-3p mimic and inhibitor in vitro.

Autophagy is an important downstream result produced by the inhibition of the mTOR signal pathway, which can be used as a new therapeutic modality for SCI as previous report [46,47,48]. The activation of mTOR showed that p-mTOR/mTOR ratio in SCI rats was significantly increased when compared with the rats from the sham group, while DG treatment could significantly inhibit mTOR activation in SCI rats. Based on these results, it is plausible that DG can activate autophagy by activating mTORC1, which may be due to the impaired flux of autophagy during SCI progression. DG treatment could rescue dysfunctional autophagy and promote autophagy after SCI in a time-dependent manner, as illustrated by analyzing the degree of autophagy at each time point (one, seven, 14, or 21 days) through p62 and LC3 expression. Rheb expression was increased in light-induced retinal neurons, and the expression of caspase-3 was enhanced, with the overlap of Rheb and caspase-3 under the detection by double-labeled fluorescent immunoassay [49]. Rheb activation not only promotes cellular proliferation and differentiation, but also enhances cellular apoptosis in response to diverse toxic stimuli. Furthermore, we also found that the expression profiles of p62 and LC3 were parallel to Rheb in a time-dependent manner. DG treatment could enhance the expression of Rheb and decrease the expression of miR-155-3p, following the significant reduction of caspase-3 and Bax expression, and up-regulation of Bcl-2 expression. However, the potential mechanisms for Rheb activation in the presence of DG still need to be explored further. Therefore it is necessary to further clarify the molecular mechanisms and the potential relationship between the inhibition of SCI-induced apoptosis, and the activation of autophagy or the rescuing of impaired flux of autophagy during DG treatment after SCI, using corresponding autophagy activators and inhibitors in vitro, as well as to examine whether miR-155-3p/Rheb is relevant to neuronal apoptosis after SCI.

The outcomes of these studies will provide a reference for uncovering the partial underlying mechanisms and exploring the therapeutic targets of SCI and other autophagy-associated diseases during intervention, using natural products.

## 4. Materials and Methods

### 4.1. Drugs and Reagents

Diosgenin glucoside (CAS No. 14144-6-0, Bellancom) was ordered from Beijing Universal Materials Co., Ltd. (Beijing, China), with the purity, as detected by high performance liquid chromatography, being greater than 98%. The major primary antibodies against the following proteins such as Bax, Bcl-2, Caspase-3, p62, Beclin1, mTOR, p-mTOR, Rheb, and p70S6K, were purchased from Cell Signaling Technology (Boston, MA, USA). Primary antibodies against LC3A/B, AKT, p-AKT and PI3K were purchased from Sigma (Foster, CA, USA). The secondary antibody was purchased from Beyotime Biotechnology Company (Shanghai, China), and fluorescent secondary antibody was purchased from Licor (Lincoln, NE, USA).

### 4.2. Animal Grouping and Treatments

A total of 120 healthy SD (Sprague Dawley) rats of SPF (Specific Pathogen Free) grade with body weights of 250 to 300 g were purchased from the Experimental Animal Center of Hubei province and housed in a temperature-controlled environment at room temperature of 20 to 24 °C and a relative humidity of 50 ± 20%. The rats were provided with standard feed and drinking water freely. All animal experiment protocols were reviewed and approved by the animal ethics committee of Hubei University for Nationalities (No. 2016(021)) in July 2016, and experimental animal welfare and ethical principles were followed.

A rat model with acute SCI was established according to the previously reported Allen’s weight-striking method [50] with minor modification. After inducing anesthesia using 10% chloral hydrate solution at a dose of 3 mL/kg, the rats were fixed on the operation table in a prone position, and sequentially subjected to hair removal and disinfection. The location of T10 was determined, and an incision with a length of 2 to 3 cm was made at the center of T10. After cutting the tissues layer by layer, the processus spinosus of T9–T11 was fully exposed. The processus spinosus and lamina of T9-T11 were removed by clamp to expose the spinal canal and spinal dura. Using a modified spinal cord striking device, a balance weight of 5 g was made to fall freely at a height of 5 cm to hit the exposed spinal dura, thus resulting in SCI of T9–T11. The occurrence of tail-spasm swing and strong lower limb contraction was used as the indicator for evaluating the successful establishment of a rat model with SCI. After the completion of the experiment, the incision wound was washed with saline, sutured, and sterilized. Then, the rats were placed in an incubator until awakening. The surgical incisions of the rats with SCI were disinfected with iodine solution twice a day for three consecutive days. Meanwhile, each rat was subjected to intramuscular injection of penicillin sodium in the hind limb at a daily dose of 40,000 U for three days. The lower abdomen was massaged with a gentle squeezing of the bladder several times each day to help with urination and defecation.

The rats were divided into the sham operation group, the SCI model group, and the DG treatment group, with 40 rats in each group. Each group was divided into four subgroups according to one, seven, 14 and 21 days (d) post-injury, respectively. The rats from the sham operation group were subjected to the exposure of the spinal cord without weight-striking damage of the spinal cord. The DG group was subjected to gavage with DG solution at a dose of 100 mg/kg, while the SCI and sham groups were subjected to the gavage with normal saline at an identical volume. The gavage was conducted according to the required time points once a day for each group.

### 4.3. Functional Evaluation

After SCI, behavioral testing for motor function was performed once a day for 21 days. Functional recovery of each rat was evaluated according to the Basso, Beattie, and Bresnahan (BBB) scale [51], which is composed of 21 different criteria for the movement of the hindlimb (from no observable hindlimb movements to normal locomotion). This scale is based on precise observation of hindlimb movements, stepping, and coordination for 4 min in an open field by a trained observer who was blind to experimental conditions.

### 4.4. Sampling

After anesthesia, five rats in each group were subjected to aorta perfusion with 0.9% saline. After the effluent was clear, perfusion with 400 mL of 4% neutral paraformaldehyde for 60 min was conducted. Then, the T9-T11 spinal cord was harvested for fixation with through 4% neutral paraformaldehyde and stored for future use. The rest of the rats within the group were perfused with 0.9% saline through the aorta. After the effluent was clear, the T9-T11 spinal cord was removed, rapidly frozen in liquid nitrogen, and then stored in a refrigerator at −80 °C for future use.

### 4.5. Hematoxylin-Eosin (HE) and Nissl Staining

Fixed spinal cord tissue was subjected to dehydration, dipping in wax, embedding, and sectioning at the thickness of 5 μm in preparation for parallel HE and Nissl staining. Continuous coronal sectioning was performed, and one slice was taken every 10 slices. The change in structure and neuronal Nissl bodies in the spinal cord was observed under a common optical microscope.

### 4.6. Immunochemistry

The wax-embedded spinal tissue in each group was sectioned into slices at a thickness of 5 µm. After wax removal, hydration and antigen repairing, the section was rinsed with PBS (phosphate buffer saline) for three times for 3 min each time. Then, the section was subjected to blocking using 10% BSA (bovine serum albumin) for 30 min. After washing with PBS 3 times for 3 min each time, the primary antibody at a dilution of 1:250 was added and incubated overnight at 4 °C. After washing with PBS three times for 3 min each time, the secondary antibodies for FITC and CY3 at a dilution of 1:1000 were incubated with the tissue slices at room temperature for 1 hr. The slices were washed with PBS 3 times for 3 min each time. DAPI staining and anti-quenching and sealing were conducted. The stained slices were subjected to the observation under a fluorescence microscope. In total, five slices were collected from each group, and three different visual fields were selected for image acquisition under a fluorescence microscope at a magnification of 400×. The slice incubated with PBS instead of primary antibody was used as the negative control. The analysis of the mean optical density (MOD) for neurons was performed using Image-pro plus 6.0 software (plus 6.0, Media Cybernetics, Bethesda, MD, USA).

### 4.7. Transmission Electron Microscopy Evaluation

The rats from different groups were sacrificed at one, three, seven, 14, and 21 days after insult. The tissue samples were collected at a point of 5 mm T9-11 spinal ventral horn. Following fixation in 2.5% (*w*/*v*) glutaraldehyde overnight, spinal cord tissues were post-fixed in 2% (*v*/*v*) osmium tetroxide and blocked with 2% (*v*/*v*) uranyl acetate. Following dehydration in a series of acetone washes, tissues were embedded in Araldite for coronal sections. Semi-thin sections and toluidine blue staining were performed for observation of location. Finally, ultra-thin sections of at least three blocks per sample were cut and observed using a Hitachi-7500 TEM (Tokyo, Japan).

### 4.8. Terminal-Deoxynucleotidyl Transferase (TdT) Mediated Nick End Labeling (TUNEL) Assay

To enable TUNEL labeling to better illustrate neuronal apoptosis of neurons, TUNEL/DAPI double labeling was performed. TUNEL staining was performed using the In Situ Cell Death Detection Kit (Beyotime, Shanghai, China. C1088) according to the manufacturer’s instructions. The number of positive cells was counted in 10 randomly chosen fields within each section. The sections per animal were respectively selected for incubation with DAPI for 3 min prior to the visualization of the sections with an optical microscope. The positive cells were identified by green TUNEL dots located in neurons with a blue nucleus at high magnification. The number of apoptotic neurons was calculated.

### 4.9. Protein Extraction from Spinal Cord Tissue and Western Blot

The spinal cord tissue was washed twice with PBS. RIPA (Radio Immunoprecipitation Assay) lysis buffer, protease inhibitor, and PMSF (Phenylmethanesulfonyl fluoride) were added to the sample. Then, the spinal cord tissue was subjected to homogenization on ice and centrifugation at 4 °C at 12,000 rpm for 15 min. Protein concentration was determined through the bicinchoninic acid (BCA) method. The proteins in the spinal cord tissue were denatured at 100 °C for 5 min. After cooling, the proteins in the samples were subjected to SDS-PAGE (Sodium dodecyl sulfate-polyacrylamide gel electrophoresis), transferred to a PVDF (Poly(vinylidene fluoride)) membrane at 275 mA for 90 min, blocked with 5% skim milk for 2 ht, and subjected to TBST (a mixture of tris-buffered saline (TBS) and Polysorbate 20 (also known as Tween 20)) washing three times for 5 min each time. Then, probing was performed with primary antibody of anti-Rheb (1:500), Bcl-2, Bax, caspase-3, AKT, p-AKT, mTOR, p-mTOR, LC3, p62, p70S6K, and β-actin (1:1000) with incubation overnight at 4 °C. Secondary antibody of HRP(Horseradish Peroxidase)-labeled goat anti-rabbit IgG(Immunoglobulin G) or HRP-labeled goat anti-mouse IgG was incubated with the membrane at room temperature for 4 hr. The images of the target proteins were developed, collected, and analyzed by the LAS4000 mini gel-imaging system.

### 4.10. Real-Time PCR of miR-155-3p

The total RNA and miRNA of collected T9-T11 spinal cord was extracted using a TRIzol extraction kit (Invitrogen, California, Calif., USA) and miRNA purification kit (Tiangen, Beijing, China) according to the manufacturer’s instructions. Primers were designed using the software Primer 5 based on the sequence information from the miRBase database (http://www.mirbase.org) and GenBank (https://www.ncbi.nlm.nih.gov/genbank/), and synthesized by Biotechnology Engineering Co., Ltd. (Shanghai, China).

The miRNA-155-3p gene primer sequence was 5′-CCTCCTACCTGTTAGCATTAAC-3′. The U6 small nuclear RNA (snRNA) was selected as the internal reference for miRNA reverse transcription and qRT-PCR. The entire amplification reaction system was performed according to the instructions of the miRNA Real-Time PCR Assay Kit (SuperReal PreMix Plus (SYBR GREEN)) (Tiangen, Beijing, China). Reverse transcription of miRNAs was conducted using cDNA synthesis of miRNA first-strand (tail addition method). The Poly(A) tail addition reaction and the cDNA synthesis reaction were carried out simultaneously (reaction system of 20 μL). In brief, 2000 ng of total RNA, 10 µL of 2× miRNA RT solution mix, and 2 µL of miRNA RT Enzyme mix were added to RNase-free water to a final total volume of 20 μL. The reaction conditions were: bathing at 37 °C bath for 60 min, and heating at 85 °C for 5 min for enzyme inactivation, and preservation at 4 °C. The obtained cDNA reaction solution was diluted 20-fold to use as the template for quantitative fluorescence detection. The qPCR reaction system (20 μL of total reaction system) contained 10 μL of 2× SuperReal PreMix Plus (with SYBR), 0.6 μL of miRNA primer Universal PCR Primer R (miR155-3P) (10 µmol/L), 0.6 μL of Universal U6 Primer F (10 μmol/L), 4 µL of miRNA first-strand cDNA obtained through reverse transcription, and 4.8 μL of RNase-free water. Reaction conditions were as follows: pre-denaturation at 95 °C for 15 min, denaturation at 95 °C for 10 s, annealing and extension at 60 °C for 30 s, and 40 reaction cycles. The samples were treated by PCR in triplicate. The relative expression of miRNA-155-3p was calculated by the 2^−ΔΔCt^ method. △Ct = Ct (miRNA) − Ct (U6), △△Ct = △Ct experiment − △Ct control. Experimental data were automatically collected and analyzed by the Mx3005P real-time fluorescence quantitative system (Agilent, Foster, CA, USA).

### 4.11. Statistical Analysis

All data were expressed as mean ± standard deviation (M ± SD). Statistical analysis was performed with GraphPad Prism 5.0 software using a one-way analysis of variance followed by a Bonferroni post-hoc test. A statistically significant difference was considered at *p* < 0.05.

## 5. Conclusions

DG can activate autophagy, down-regulate miR-155, and activate the Rheb/mTOR signal pathway, thereby alleviating spinal cord injury, promoting functional recovery of the spinal cord, and providing a theoretical reference for the treatment of spinal cord injury and the development and utilization of natural products for health promotion and neurodegenerative disease treatment. However, its exact mechanisms still need to be further explored.

## References

[1] Cripps R.A., Lee B.B., Wing P., Weerts E., Mackay J., Brown D. (2011). A global map for traumatic spinal cord injury epidemiology: Towards a living data repository for injury prevention. Spinal Cord.

[2] Chen M., Xia X., Zhu X., Cao J., Xu D., Ni Y., Liu Y., Yan S., Cheng X., Liu Y. (2014). Expression of SGTA correlates with neuronal apoptosis and reactive gliosis after spinal cord injury. Cell Tissue Res..

[3] Zhao J., Chen C., Xiao J.R., Wei H.F., Zhou X.H., Mao X.X., Zhang W.D., Qian R., Chen X.L., He M.Q. (2015). An Up-regulation of IRF-1 After a Spinal Cord Injury: Implications for Neuronal Apoptosis. J. Mol. Neurosci..

[4] Zhang Y.B., Li S.X., Chen X.P., Yang L., Zhang Y.G., Liu R., Tao L.Y. (2008). Autophagy is activated and might protect neurons from degeneration after traumatic brain injury. Neurosci. Bull..

[5] Sekiguchi A., Kanno H., Ozawa H., Yamaya S., Itoi E. (2012). Rapamycin promotes autophagy and reduces neural tissue damage and locomotor impairment after spinal cord injury in mice. J. Neurotrauma.

[6] Chen H.C., Fong T.H., Hsu P.W., Chiu W.T. (2013). Multifaceted effects of rapamycin on functional recovery after spinal cord injury in rats through autophagy promotion, anti-inflammation, and neuroprotection. J. Surg. Res..

[7] Yan W., Zhang H., Bai X., Lu Y., Dong H., Xiong L. (2011). Autophagy activation is involved in neuroprotection induced by hyperbaric oxygen preconditioning against focal cerebral ischemia in rats. Brain Res..

[8] Kanno H., Ozawa H., Sekiguchi A., Yamaya S., Itoi E. (2011). Induction of autophagy and autophagic cell death in damaged neural tissue after acute spinal cord injury in mice. Spine.

[9] Walker C.L., Walker M.J., Liu N.K., Risberg E.C., Gao X., Chen J., Xu X.M. (2012). Systemic bisperoxovanadium activates Akt/mTOR, reduces autophagy, and enhances recovery following cervical spinal cord injury. PLoS ONE.

[10] Hao H.H., Wang L., Guo Z.J., Bai L., Zhang R.P., Shuang W.B., Jia Y.J., Wang J., Li X.Y., Liu Q. (2013). Valproic acid reduces autophagy and promotes functional recovery after spinal cord injury in rats. Neurosci. Bull..

[11] Andorfer C.A., Necela B.M., Thompson E.A., Perez E.A. (2011). MicroRNA signatures: Clinical biomarkers for the diagnosis and treatment of breast cancer. Trends Mol. Med..

[12] Cheung H.H., Davis A.J., Lee T.L., Pang A.L., Nagrani S., Rennert O.M., Chan W.Y. (2011). Methylation of an intronic region regulates miR-199a in testicular tumor malignancy. Oncogene.

[13] Dellago H., Preschitz-Kammerhofer B., Terlecki-Zaniewicz L., Schreiner C., Fortschegger K., Chang M.W., Hackl M., Monteforte R., Kühnel H., Schosserer M. (2013). High levels of oncomiR-21 contribute to the senescence-induced growth arrest in normal human cells and its knock-down increases the replicative lifespan. Aging Cell.

[14] Jovicic A., Jolissaint J.F.Z., Moser R., Silva Santos M. de F., Luthi-Carter R. (2013). MicroRNA-22 (miR-22) overexpression is neuroprotective via general anti-apoptotic effects and may also target specific Huntington’s disease-related mechanisms. PLoS ONE.

[15] Faraoni I., Antonetti F.R., Cardone J., Bonmassar E. (2009). miR-155 gene: A typical multifunctional microRNA. Biochim. Biophys. Acta.

[16] Wu J., Lu C., Diao N., Zhang S., Wang S., Wang F., Gao Y., Chen J., Shao L., Lu J. (2012). Analysis of microRNA expression profiling identifies miR-155 and miR-155 as potential diagnostic markers for active tuberculosis: A preliminary study. Hum. Immunol..

[17] Yang K., Wu M., Li M., Li D., Peng A., Nie X., Sun M., Wang J., Wu Y., Deng Q. (2014). miR-155 suppresses bacterial clearance in Pseudomonas aeruginosa-induced keratitis by targeting Rheb. J. Infect. Dis..

[18] Wang J., Yang K., Zhou L., Wu M., Wu Y., Zhu M., Lai X., Chen T., Feng L., Li M. (2013). MicroRNA-155 promotes autophagy to eliminate intracellular mycobacteria by targeting Rheb. PLoS Pathog..

[19] Zhang C., Liu X.R., Cao Y.C., Tian J.L., Zhen D., Luo X.F., Wang X.M., Tian J.H., Gao J.M. (2017). Mammalian target of rapamycin/eukaryotic initiation factor 4F pathway regulates follicle growth and development of theca cells in mice. Reprod. Fertil. Dev..

[20] Li Y., Corradetti M.N., Inoki K., Guan K.L. (2004). TSC2: Filling the GAP in the mTOR signaling pathway. Trends Biochem. Sci..

[21] Inoki K., Li Y., Xu T., Guan K.L. (2003). Rheb GTPase is a direct target of TSC2 GAP activity and regulates mTOR signaling. Genes Dev..

[22] Ono M., Yanai Y., Ikeda T., Okawa M., Nohara T. (2003). Steroids from the underground parts of Trillium kamtschaticum. Chem. Pharm. Bull. (Tokyo).

[23] Ono M., Sugita F., Shigematsu S., Takamura C., Yoshimitsu H., Miyashita H., Ikeda T., Nohara T. (2007). Three new steroid glycosides from the underground parts of Trillium kamtschaticum. Chem. Pharm. Bull. (Tokyo).

[24] Wu H., Qiu Y., Shu Z., Zhang X., Li R., Liu S., Chen L., Liu H., Chen N. (2016). Protective effect of Trillium tschonoskii saponin on CCl4-induced acute liver injury of rats through apoptosis inhibition. Can. J. Physiol. Pharmacol..

[25] Jiang G., Yu X., Xu K. (2013). Analysing on aging model rats by injecting d-gal into abdominal cavity and Subcutaneous. Chin. J. Gerontol..

[26] Chen X., Zhu M., Qin F. (2015). Effect of extract of Trillium tschonoskii Maxim on ciliary neurotropic factor and its receptor α in rats suffering from spinal cord injury. Med. J. Chin. People’s Lib. Army.

[27] Wang L., Du J., Zhao F., Chen Z., Chang J., Qin F., Wang Z., Wang F., Chen X., Chen N. (2018). Trillium tschonoskii maxim saponin mitigates D-galactose-induced brain aging of rats through rescuing dysfunctional autophagy mediated by Rheb-mTOR signal pathway. Biomed. Pharmacother..

[28] Campolo M., Esposito E., Ahmad A., Di Paola R., Wallace J.L., Cuzzocrea S. (2013). A hydrogen sulfide-releasing cyclooxygenase inhibitor markedly accelerates recovery from experimental spinal cord injury. FASEB J..

[29] Yin Y.M., Lu Y., Zhang L.X., Zhang G.P., Zhang Z.Q. (2015). Bone marrow stromal cells transplantation combined with ultrashortwave therapy promotes functional recovery on spinal cord injury in rats. Synapse.

[30] Bai L., Mei X., Shen Z., Bi Y., Yuan Y., Guo Z., Wang H., Zhao H., Zhou Z., Wang C. (2017). Netrin-1 Improves Functional Recovery through Autophagy Regulation by Activating the AMPK/mTOR Signaling Pathway in Rats with Spinal Cord Injury. Sci. Rep..

[31] Chen J., Cui Z., Li W., Shen A., Xu G., Bao G., Sun Y., Wang L., Fan J., Zhang J. (2013). MCM7 expression is altered in rat after spinal cord injury. J. Mol. Neurosci..

[32] Hu J.Z., Huang J.H., Zeng L., Wang G., Cao M., Lu H.B. (2013). Anti-apoptotic effect of microRNA-21 after contusion spinal cord injury in rats. J. Neurotrauma.

[33] Filip A. (2007). MiRNA—New mechanisms of gene expression control. Postepy Biochem..

[34] Liu D.Z., Tian Y., Ander B.P., Xu H., Stamova B.S., Zhan X., Turner R.J., Jickling G., Sharp F.R. (2010). Brain and blood microRNA expression profiling of ischemic stroke, intracerebral hemorrhage, and kainate seizures. J. Cereb. Blood Flow Metab..

[35] Greco R., Demartini C., Zanaboni A.M., Blandini F., Amantea D., Tassorelli C. (2018). Endothelial nitric oxide synthase inhibition triggers inflammatory responses in the brain of male rats exposed to ischemia-reperfusion injury. J. Neurosci. Res..

[36] Xu L., Leng H., Shi X., Ji J., Fu J., Leng H. (2017). MiR-155 promotes cell proliferation and inhibits apoptosis by PTEN signaling pathway in the psoriasis. Biomed. Pharmarcother..

[37] Liu Y., Pan Q., Zhao Y., He C., Bi K., Chen Y., Zhao B., Chen Y., Ma X. (2015). MicroRNA-155 Regulates ROS Production, NO Generation, Apoptosis and Multiple Functions of Human Brain Microvessel Endothelial Cells Under Physiological and Pathological Conditions. J. Cell Biochem..

[38] Wan G., Xie W., Liu Z., Xu W., Lao Y., Huang N., Cui K., Liao M., He J., Jiang Y. (2014). Hypoxia-induced MIR155 is a potent autophagy inducer by targeting multiple players in the MTOR pathway. Autophagy.

[39] Brown H.L., Kaun K.R., Edgar B.A. (2012). The small GTPase Rheb affects central brain neuronal morphology and memory formation in Drosophila. PLoS ONE.

[40] Gracias N.G., Shirkey-Son N.J., Hengst U. (2014). Local translation of TC10 is required for membrane expansion during axon outgrowth. Nat. Commun..

[41] Gu T., Zhao T., Hewes R.S. (2014). Insulin signaling regulates neurite growth during metamorphic neuronal remodeling. Biol. Open.

[42] Gregory E.N., Codeluppi S., Gregory J.A., Steinauer J., Svensson C.I. (2010). Mammalian target of rapamycin in spinal cord neurons mediates hypersensitivity induced by peripheral inflammation. Neuroscience.

[43] Zou J., Zhou L., Du X.X., Ji Y., Xu J., Tian J., Jiang W., Zou Y., Yu S., Gan L. (2011). Rheb1 is required for mTORC1 and myelination in postnatal brain development. Dev. Cell.

[44] Cao M., Tan X., Jin W., Zheng H., Xu W., Rui Y., Li L., Cao J., Wu X., Cui G. (2013). Upregulation of Ras homolog enriched in the brain (Rheb) in lipopolysaccharide-induced neuroinflammation. Neurochem. Int..

[45] Hartman N.W., Lin T.V., Zhang L., Paquelet G.E., Feliciano D.M., Bordey A. (2013). mTORC1 targets the translational repressor 4E-BP2, but not S6 kinase 1/2, to regulate neural stem cell self-renewal in vivo. Cell Rep..

[46] Yu Q.J., Yang Y. (2016). Function of SOD1, SOD2, and PI3K/AKT signaling pathways in the protection of propofol on spinal cord ischemic reperfusion injury in a rabbit model. Life Sci..

[47] Jung S.Y., Kim D.Y., Yune T.Y., Shin D.H., Baek S.B., Kim C.J. (2014). Treadmill exercise reduces spinal cord injury-induced apoptosis by activating the PI3K/Akt pathway in rats. Exp. Ther. Med..

[48] Don A.S., Tsang C.K., Kazdoba T.M., D’Arcangelo G., Young W., Zheng X.F. (2012). Targeting mTOR as a novel therapeutic strategy for traumatic CNS injuries. Drug Discov. Today.

[49] Shu Q., Xu Y., Zhuang H., Fan J., Sun Z., Zhang M., Xu G. (2014). Ras homolog enriched in the brain is linked to retinal ganglion cell apoptosis after light injury in rats. J. Mol. Neurosci..

[50] Gruner J.A. (1992). A monitored contusion model of spinal cord injury in the rat. J. Neurotrauma.

[51] Fournier A.E., Takizawa B.T., Strittmatter S.M. (2003). Rho kinase inhibition enhances axonal regeneration in the injured CNS. J. Neurosci..

